# The Utility of Verbal Therapy for Pediatric Cancer Patients and Survivors: Expressive Writing, Video Narratives, and Bibliotherapy Exercises

**DOI:** 10.3389/fped.2021.579003

**Published:** 2021-02-04

**Authors:** Jill K. Jones, John F. Evans, Raymond C. Barfield

**Affiliations:** ^1^Trinity College of Arts & Sciences, Duke University, Durham, NC, United States; ^2^Duke Integrative Medicine, Durham, NC, United States; ^3^Memorial Health University Medical Center (Palliative Care), Savannah, GA, United States

**Keywords:** expressive writing, video narratives, bibliotherapy, pediatric cancer, resilience, well-being

## Abstract

Childhood cancer is a stressful experience. No pediatric patient, however, should be made to feel as if their concerns and feelings about their cancer experience must be bottled up inside. Importantly, talking and writing about one's illness has myriad implications for young cancer patients and survivors. The most salient of these may include increased understanding of one's condition as well as improved physical and cognitive symptoms (e.g., lowered depression, decreased anxiety, and an enhanced quality of life overall). This literature review explores three promising avenues for verbal therapy in the pediatric oncology setting: expressive writing, video narratives, and bibliotherapy exercises. Several recent studies, covering verbal therapy methods from illness blogging to book interventions, are referenced and discussed. Ultimately, we conclude that expressive writing, video narratives, and bibliotherapy exercises are valuable, feasible, inexpensive, and acceptable tools for patients and survivors of childhood cancer to facilitate self-expression—and to find meaning in the uncertainty and anxiety that cancer inherently fosters. We recommend that future studies investigate this theme so that we may improve quality of life and mental health for pediatric cancer patients and survivors worldwide.

## Introduction

In 2005, a review by Patenaude and Kupst found that up to 20% of pediatric cancer survivors experience moderate-to-severe posttraumatic stress due to their illness. A much larger percentage of pediatric cancer patients struggles with academics, social relationships, and self-esteem during and after their cancer treatment (1). In facing these challenges, perhaps the most useful tool is one that almost all of us have ready access to: our own language.

*Verbal therapy*, or expressing one's self through words, can help pediatric cancer patients and survivors find closure and foster personal growth. Here, we review the current literature on the utility of three promising forms of verbal therapy—expressive writing, video narratives, and bibliotherapy exercises—in pediatric oncology. First, we present a summary of each form. Then, we differentiate these forms from the more traditional “talk therapy” (traditional psychotherapy), synthesize the findings of 12 most relevant studies (Table 1), and discuss implications for their broader use with pediatric cancer patients and survivors. By encouraging individuals to process their deepest thoughts and feelings about their illness experience (specifically in narrative form), we argue that expressive writing, video narratives, and bibliotherapy exercises can positively impact feelings of stress and anxiety, interpersonal relationships, and overall quality of life for this subpopulation.

**Table 1:** Studies explored in-depth in this literature review.

**Study**	**Genre**	**Country**	**Modality**	**Relevant one-sentence summary**
Anzeneder et al. (2018)	Exploratory intervention	Italy	Expressive writing	Expressive writing had positive effects on perceived quality of life, internalized symptoms, and coping skills for three out of four adolescent brain tumor patients in the study.
Chaparro (2)	Exploratory intervention	Canada	Expressive writing, psychotherapy-influenced interview	Pediatric cancer survivors completed psychotherapy-influenced phone interviews while many of their mothers completed expressive writing exercises, illuminating positive correlations between meaning making, “cancer talk” frequency, distress-related disclosure, and posttraumatic growth.
Keim-Malpass et al. (3)	Exploratory qualitative analysis	USA	Expressive writing (illness blogs)	Thematic analysis of seven adolescent cancer blogs granted personal insight into unique cancer experiences while pointing to the creative and psychosocial benefits of writing about illness.
Crook and Love (4)	Exploratory qualitative analysis	USA	Expressive writing (online support groups)	Qualitative analysis of messages in an online cancer support group elucidated both disadvantages (spreading medical misinformation, lack of synchronous communication) and advantages (space for venting healthcare frustrations and connecting with other patients) of such expressive writing.
Akard et al. (5)	Randomized, controlled intervention	USA	Video narrative construction (digital storytelling)	Analyzing results with 15 experimental group and 13 control group participants, this study determined that a legacy-making, digital storytelling intervention for children with cancer was feasible and fostered improvement in emotional and school functioning.
Wilson et al. (6)	Literature review	USA	Video narrative construction (digital storytelling)	A synthesis of findings from 64 publications, related to storytelling and pediatric cancer, concluded that digital storytelling can be an especially useful tool to promote social development, cultural congruence, self-discovery, and self-understanding.
Pereira (7)	Controlled intervention	USA	Video narrative construction	Completion of a video therapy intervention resulted in significant positive correlations with cancer patients' health-related behaviors, personal relationships with individuals on their medical teams, their perceived impact on others, and a sense of resilience and clarity regarding their illness experience.
Pereira et al. (8)	Qualitative analysis	USA	Video narrative construction	Crafting a video narrative helped an adolescent leukemia patient gain greater clarity about the overarching impact of cancer on his life and a more positive attitude about accomplishing future goals.
Pereira et al. (9)	Exploratory intervention	USA	Video narrative construction	After examining content from video testimonials of 25 adolescent and young adult cancer patients/survivors, video narratives seem to be an effective means through which to explore thoughts and feelings about the youth cancer experience.
Schneider (2012)	Exploratory intervention	USA	Bibliotherapy	Reading a disease-relevant story, entitled *Nikki's Day at Chemo*, correlated with improved perceptions of intrapersonal functioning and decreased physiological arousal among a cohort of pediatric participants with cancer.
Thurneck et al. (10)	Book chapter	USA	Bibliotherapy	Bibliotherapy is a viable therapeutic approach for young people with disabilities and healthcare needs, as it can help them (a) understand their condition in a language comprehensible to them and (b) see that they are not alone.
Malibiran et al. (11)	Literature review	USA	Bibliotherapy	An in-depth scrutiny of nine recent studies regarding bibliotherapy provided preliminary evidence for its potential to mitigate symptoms of depression, ineffective coping, and anxiety among cancer patients.

### Expressive Writing

**Figure 1:**
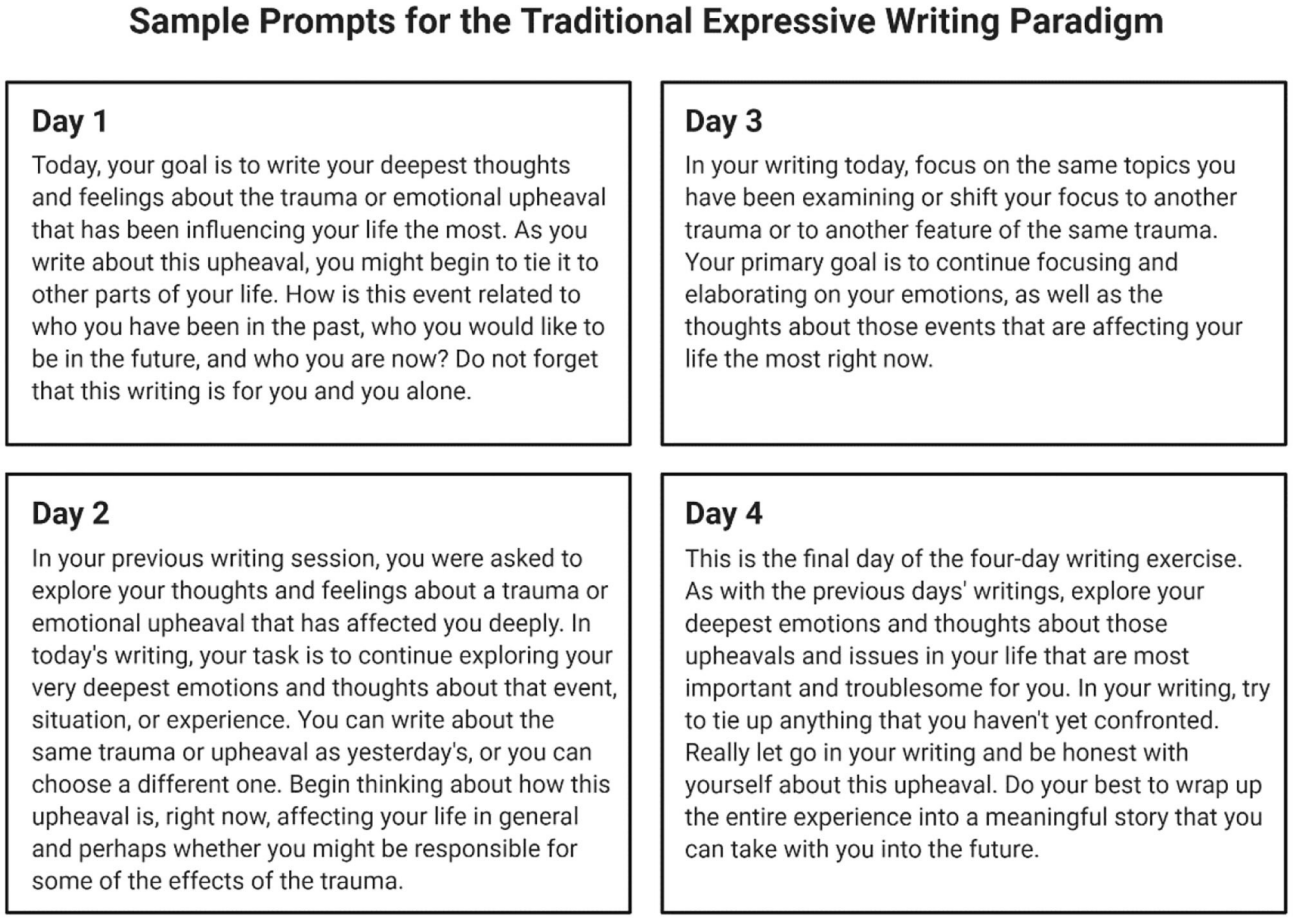
Sample prompts for the traditional 4-day expressive writing paradigm, asking participants to explore their deepest thoughts and feelings about a traumatic event or challenging experience. Adapted from Pennebaker and Evans (13) and created using BioRender.com.

Expressive writing (EW) was pioneered by Pennebaker in the early 1980s (12). The traditional EW paradigm (Figure 1) lasts 4 days, asking participants to reflect on one trauma-centered writing prompt for approximately 20 min/day (13). Across a variety of populations, many studies have demonstrated strong correlations between EW and lower levels of depression, lower levels of pain and medication use, and a positive, stable increase in mood (14, 15). In a meta-analysis of 13 EW studies, Smyth (16) examined consequent outcomes for physical health, psychological well-being, physiological functioning, and general functioning and found a strong, significant effect size (*d* = 0.47, *p* < 0.001).

Excitingly, EW has shown particular promise with specific subpopulations of individuals, such as pediatric cancer patients and survivors. These include, but are not limited to the following: individuals with irritable bowel syndrome (17), postdeployment military couples (18), women with a history of childhood sexual abuse (19), and women with breast cancer (20–25). In later sections of this review, we evaluate four studies on EW and its related forms (narrative writing, illness blogs, and online illness support groups) in the pediatric oncology setting.

### Video Narratives

A video narrative is a recording of someone talking about an influential event or situation in their life. This recorded story can later be played back, reflected upon, and/or shared with other people on a video-sharing platform (e.g., YouTube). Synonyms for video narratives, a relatively new therapeutic approach, include video testimonials, testimonial videos, and therapeutic film-making (7, 8). Here, we review five publications on the use of video narratives for self-reflection among pediatric cancer patients and survivors.

### Bibliotherapy Exercises

Dating back to ancient Greece, bibliotherapy is defined as the use of books as therapy in the context of mental or physical illness (26). Clients are assigned or offered reading material that is highly relevant to their own personal challenges, ranging from self-help books to memoirs written by individuals with similar backgrounds. Recent studies suggest that bibliotherapy can promote myriad positive effects for young people facing hardship. These range from increased empathy among aggressive elementary school children (27) to improvements in self-concept and peer perceptions among average elementary schoolers (28) to clinically significant changes in anxiety severity among children with nighttime fears (29). In this review, we discuss Schneider's seminal 2012 study on bibliotherapy for children with cancer, bolstered by support from two other publications on bibliotherapy with various cancer patient populations.

### Talk Therapy

In talk therapy, or traditional psychotherapy, a mental health professional asks probing questions and engages in thought-provoking conversation with a client to help them address an issue in their life (30). Contemporary randomized controlled trials (RCTs) suggest that talk therapy can significantly reduce feelings of anxiety, depression, and other symptoms of psychological distress among adult cancer patients (31–33). Although a dearth of RCTs on talk therapy for pediatric cancer patients and survivors exists, several case vignettes (34, 35), reports on adolescent cancer support groups (36, 37), and studies on psychotherapy-influenced interviews (38–40) with this subpopulation have been conducted. Taken together, these studies demonstrate that for younger cancer patients and survivors, talking with others about their disease can help them come to terms with their cancer experience and form meaningful social bonds.

However, there are several caveats to talk therapy's widespread acceptance and accessibility. In 2019, Nitzburg and Farber (41) surveyed 267 adult clients of Talkspace (a text-based, online psychotherapy initiative) about perceived barriers to engaging in traditional, face-to-face talk therapy. Of respondents, 54.2% reported that traditional talk therapy is too expensive (often ~$100/1-h session), and 38.3% indicated that this verbal therapy is not covered by their insurance; 42.1% also reported that traditional talk therapy is too time-consuming or inconvenient (e.g., including time traveled to and from their therapist's office), while 27.9% said they had never found talk therapy particularly helpful. It is our firm belief that EW, video narrative production, and bibliotherapy overcome these issues. In contrast to traditional talk therapy, these verbal therapies can be conducted on patients' own time, at little to no cost, and with very few resources (e.g., a pen and paper, a camera, and/or books). Children and teens may find them especially appealing due to their focus on creative and open storytelling, as well as their novelty; EW, video narrative production, and bibliotherapy are not as well known nor as well studied as traditional psychotherapy. For these reasons, we have chosen to focus on EW, video narrative production, and bibliotherapy for pediatric cancer patients and survivors over traditional talk therapy in this review.

## Methods

The following terms were searched on Google Scholar, yielding the following results as of 16 January 2020:

- “expressive writing” “pediatric cancer”−108 results, 6 selected
- “expressive writing” “pediatric patients”−84 results, 7 selected
- “expressive writing” “children” “disease”−4,110 results, 1 selected
- “bibliotherapy” “pediatric cancer patients”−55 results, 4 selected
- “childhood cancer” “expressive writing”−141 results, 8 selected.

**Figure 2:**
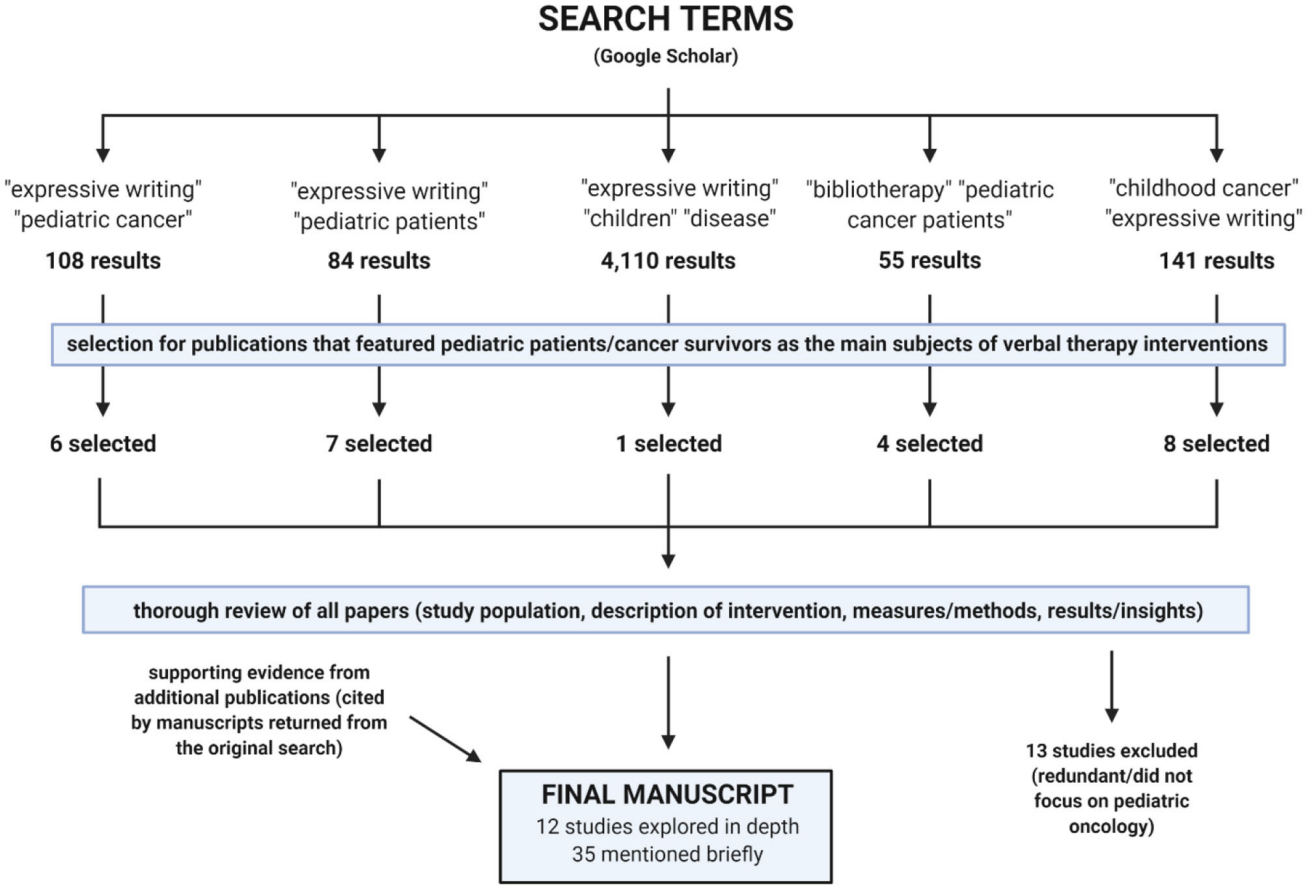
Process of review for publications included in this manuscript. Created using BioRender.com.

Only papers that featured pediatric patients and/or cancer survivors as their main subjects of verbal therapy interventions were selected (Figure 2). These papers were reviewed according to the following categories: *study population* (if applicable), *description of intervention* (if applicable), *measures/methods* (if applicable), and *results/insights*. Studies were analyzed for possible grouping themes, which ultimately emerged as talk therapy, expressive writing, video narratives, and bibliotherapy. Due to their novelty, lower cost, and higher possibility for acceptance and accessibility, we chose to focus on expressive writing, video narratives, and bibliotherapy in our final review.

Overall, half (13/26) of the originally analyzed papers were ultimately excluded from our review as we adopted this narrower focus. Thirty-four other papers, either cited in the manuscripts above or suggested by the reviewers of this manuscript, were consequently analyzed and discussed. All provide supporting evidence for the utility of expressive writing, video narratives, and bibliotherapy exercises in our specific patient/survivor population of interest (pediatric oncology).

## Results

### Expressive Writing and Related Forms

In 2018, Anzeneder et al. (48) conducted an EW study with four teenage brain tumor patients. The intervention consisted of four writing sessions, spaced 1 week apart, and participants completed three questionnaires (the Child Behavior Checklist, the Pediatric Quality of Life Inventory, and the Adolescent Coping Orientation for Problem Experiences) before, immediately after, and 1 month following its conclusion. Alongside data collected *via* the Linguistic Inquiry Word Count Program (which assesses the frequency of positive- or negative-affect words used by participants, like *resilient* or *depressed*), scores from these questionnaires helped quantify EW's effects on patients' coping skills, internalized symptoms, and perceived quality of life. Ultimately, the researchers found positive trends in each one of these variables for three out of four EW intervention participants. Given the extremely small sample size, replication studies with larger patient cohorts must be completed. At least for some pediatric cancer patients, however, Anzeneder et al.'s (2018) work does point to EW as an inexpensive, feasible, and meaningful verbal therapy.

In 2015, Chaparro (2) completed a similar study with 100 young adult survivors of childhood cancer and 88 of their mothers. First, Chaparro asked the 100 survivors to participate in a 20-min phone interview about their life story. Each participant was encouraged to think of their life as a book or as a novel and to identify key turning points therein. Pre- and postintervention responses on relevant questionnaires (assessing attachment style, posttraumatic growth, and psychosocial adjustment) were scored and compared, and all interview responses were coded for meaning making and coherence. Second, Chaparro asked the 88 mothers in the study to complete an EW task about a particularly challenging moment in their child's cancer experience. Before and after this task, each mother completed questionnaires about distress-related disclosure, “cancer talk” frequency, and dispositional optimism.

Overall, Chaparro found that meaning making was higher for cancer-related than for noncancer-related turning points of survivors' narratives. Mothers' “cancer talk” frequency was positively associated with this meaning making, and mothers' distress-related disclosure also positively correlated with survivors' posttraumatic growth. For pediatric cancer patients, survivors, and caregivers, Chaparro posits that verbalizing one's cancer narrative can enhance psychological well-being, and she encourages future studies to be conducted on links between appropriate themes.

In line with this suggestion, Keim-Malpass et al. (3) recently explored seven illness blogs (focused on cancer disease progression) curated by self-identified adolescents with cancer (AWC). Using both *a priori* coding strategies and *post hoc* observations, key themes that emerged across these blogs included normalizing news, facing treatment failure, and reconciling *chronos* or treating time as an entity of which patients did not have enough. In normalizing news, the researchers noted that AWC often turned to self-deprecating humor and/or pragmatism when describing turning points in their cancer story (e.g., “I've been coughing [more] and whatever”; “It's back. My remission is over.”). In facing treatment failure, AWC described profound transitions from periods of uncertainty into more active decision-making in their treatment plans (e.g., choosing to refuse or accept another round of chemotherapy). In reconciling *chronos*, patients tended to treat time like currency, hoping to buy more of it through palliative care options. Overall, Keim-Malpass et al. (3) conclude that each illness blog was an unparalleled outlet for its author to assert agency over their illness experience and tell their narrative in their own words. They assert that such EW-focused blogs are not just tools for patients' self-expression but also opportunities for healthcare professionals to understand the cancer experience through a patient's eyes—and to adjust their care accordingly. In the future, it would be interesting to explore how the themes that Keim-Malpass et al. (3) identified manifest among participants in other studies, as well as how we might tailor verbal therapy exercises to encourage discussion and reflection about them.

In 2017, Crook and Love (4) further explored themes across EW-inspired cancer blogs. Specifically, they examined a *collective* illness blog, evaluating advantages and disadvantages to participating in an online cancer support group. This group was frequented by over 6,000 AWC affected by cancer at any stage (diagnosis, treatment, or remission) and featured more than 20,000 posts discussing self-advocacy, frustration with the healthcare system, and when/how to disclose illness with others. A few disadvantages that the authors identified were both the potential to spread medical misinformation and the lack of synchronous communication (some posts can go uncommented upon or unanswered for months). However, a large advantage was that such collective, online EW offers necessary space for patients to write about their cancer experience and identify with others undergoing similar treatment regimens. EW, whether collective or not, can often be an unmatched avenue for cultivating self-acceptance and self-disclosure in pediatric oncology, and the studies from Anzeneder et al. (2018), Chaparro (2), Keim-Malpass et al. (3), and Crook and Love (4) support this in myriad, albeit sometimes subtle, ways.

### Video Narratives

For some younger patients—especially those who are not yet literate or who may struggle to read and write (due to lethargy, “chemo fog,” or other side effects of cancer treatment)—crafting video narratives may be a more appealing, yet equally as intriguing, option as EW for verbal therapy. In early 2015, two publications from Akard et al. (5) and Wilson et al. (6) attested to the power of digital storytelling. For their study, Akard et al. (5) completed a randomized video therapy intervention with 28 pediatric cancer patients; 15 answered questions about legacy-making and created a digital story about themselves, while the remaining 13 served as a control group (not answering any questions nor crafting a digital story with photographs, music, and video clips of themselves speaking). After the intervention, children in the experimental group reported slightly better emotional and school functioning than controls, and all experimental participants reported that the digital storytelling activity was enjoyable and fun. For their study, Wilson et al. (6) conducted a literature review of 64 publications at the intersection between storytelling and pediatric oncology. Ultimately, they concluded that digital storytelling, in particular, is a promising, modern approach to foster self-understanding, social development, cultural congruence, and self-discovery among pediatric cancer patients.

Pereira has substantiated the findings of the above studies with evidence from her video therapy work in pediatric oncology. In her first study (2017), Pereira (7) recruited adolescents with and without cancer to produce video narratives and answer questions about the videos they made. AWC were instructed to talk to the camera about their cancer experience, while control participants were asked to discuss a personal hardship they had faced in their life. Intriguingly, Pereira found that controls tended to address this personal hardship in a negative light, while AWC often referred to “silver linings” in their cancer experience. By offering an opportunity for AWC to recite their entire cancer story, video therapy brought a unique sense of resilience, clarity, and increased understanding of AWC's cancer experience overall. Significant positive correlations between reciting a personal narrative and participants' health-related behaviors, personal relationships with participants' medical teams, and participants' perceived impact on others were revealed.

For her second study (2017), Pereira et al. (8) filmed an adolescent leukemia patient as he recited his personal story since being diagnosed. Talking most in-depth about his access to quality medical care, the impact of cancer treatment on his overall health, his legacy as a cancer patient, and cancer's effects on his personal relationships, the patient was able to contextualize and come to a conclusion about his cancer story through video therapy. In the patient's own words, this video narrative exercise helped him gain greater clarity about the overarching impact of cancer on his life, as well as a more positive attitude about accomplishing future goals.

In a recent study (2020), Pereira et al. (9) asked nine survivors and 16 current patients of adolescent cancer to record a private video about their cancer experience. Content analysis identified self-esteem, missing social events, discomfort with the children's hospital environment, and frustration with the way healthcare providers spoke to them as predominant themes. Video therapy helped participants express their deepest feelings about such personal topics—and, in the future, could help healthcare providers learn how they can better serve AWC.

### Bibliotherapy Exercises

As a third form of verbal therapy, bibliotherapy may be most appealing to shier/more reserved pediatric cancer patients, who wish to engage in a more passive activity: finding their own voice by reading about others with similar lived experiences. In 2012, Schneider (49) published her doctoral dissertation on a bibliotherapy intervention with 21 pediatric cancer patients. Participants were read the book *Nikki's Day at Chemo* and asked how they might apply some of the coping strategies discussed in the book to their own lives (for example, using one's imagination to cope with nervousness).

Postintervention, participants reported both high satisfaction with the book and statistically significant improvement within the measure of intrapersonal functioning. One child told Schneider: “having you read this book to me makes me want to write my own story that everyone can read,” and one parent reported that it inspired her son to “speak more freely about his disease” with his healthcare team, his friends at school, and the general public. Schneider concludes that bibliotherapy is an inexpensive, convenient, and useful psychological intervention for pediatric cancer patients, and her findings are supported by publications on bibliotherapy for pediatric patients (10) and cancer patients (11) more generally.

In their 2007 book, Thurneck et al. highlight two key advantages of bibliotherapy for pediatric patients, either with cancer or without. One is that bibliotherapy can help pediatric patients understand their illness in a language comprehensible to them (e.g., with simpler words and with accompanying illustrations). The second is that illness-related intervention books can help patients with disabilities and/or who are critically ill see that they are not alone, especially if they use a lot of imagery and illustrations. Notably, cancer treatment often affects physical appearance, and body image difficulties are found across patients with diverse cancer sites (42). Bibliotherapy is one way to validate and address these challenges.

In support of this, Malibiran et al. (11) conducted a recent literature review of nine bibliotherapy interventions with a variety of cancer patients. (One of these interventions was Schneider's 2012 study.) Excitingly, the authors found that all nine interventions produced positive effects on patient variables including coping skills, anxiety, depression, self-esteem, and interpersonal quality of life. Although the reviewed interventions largely took place with adults (rather than pediatric patients), these reportedly uniform benefits may suggest high efficacy and acceptability of bibliotherapy among cancer patients overall. More studies—with significantly larger sample sizes—should evaluate the effectiveness of bibliotherapy for pediatric cancer patients in decreasing feelings of stress, anxiety, depression, fear, and isolation.

## Discussion

Cancer is not just a physical illness; for many patients, it disrupts personal relationships, body image, mood regulation, and psychological well-being as well (43, 44). For pediatric cancer patients—who may experience a total loss of normal childhood—these challenges may be even more pronounced. Encouragingly, EW, video narratives, and bibliotherapy exercises work to validate and address such cancer-related difficulties. By approaching the illness experience through a narrative lens, these verbal therapies encourage patients to share their deepest thoughts and feelings about cancer and its broad impact on their lives. In contrast to traditional talk therapy (psychotherapy), these verbal therapies can also be delivered at little to no cost, anywhere, and completely on patients' own time. Here, we have reviewed twelve main studies on the utility of EW, video narratives, and bibliotherapy exercises in pediatric oncology. We urge future studies to further examine the efficacy of these techniques for pediatric cancer patients and survivors.

Naturally, however, a few limitations must be acknowledged. First, every study discussed in this review featured a relatively small sample size (*n* ≤ 100). Until further research is done, this small scope limits the generalizability of conclusions made about EW, video narratives, and bibliotherapy exercises in pediatric oncology. Second, not every study reviewed here was randomized and controlled nor were measures for specific outcomes (e.g., resilience) consistent across studies. In order to draw more robust conclusions about the effects that EW, video narrative construction, and bibliotherapy may have on depression, anxiety, and other psychological symptoms, outcome measures (i.e., measures that allow the efficacy of EW, video therapy, and/or bibliotherapy to be quantifiably assessed) must be standardized. These scorable measures may range from behavioral checklists to depression and anxiety questionnaires, but researchers must come to consensus on which ones must be utilized and reported in every study. This standardization will allow for more direct comparison of these verbal therapies' benefits across studies, environments, and even individuals. Not to be forgotten, such questionnaires and checklists must also all be appropriately scaled and written at appropriate reading levels for children and adolescents, rather than adults. Finally, it is important to acknowledge that EW, video therapy, and/or bibliotherapy may not be an effective therapeutic approach for every child or adolescent with cancer. Pennebaker and Evans (13) warn that re-exposure to a traumatic or challenging experience (like cancer treatment), either by writing or talking about it, may become overwhelming for some individuals. Healthcare teams, caregivers, and pediatric patients and survivors themselves should work together in weighing the potential long-term benefits of these verbal therapies against the feelings of distress they might initially provoke. In any case, careful consideration should be taken in determining what verbal therapy form may best suit each individual patient/survivor, as well as at which stage of their illness experience verbal therapies should/could be introduced.

**Figure 3:**
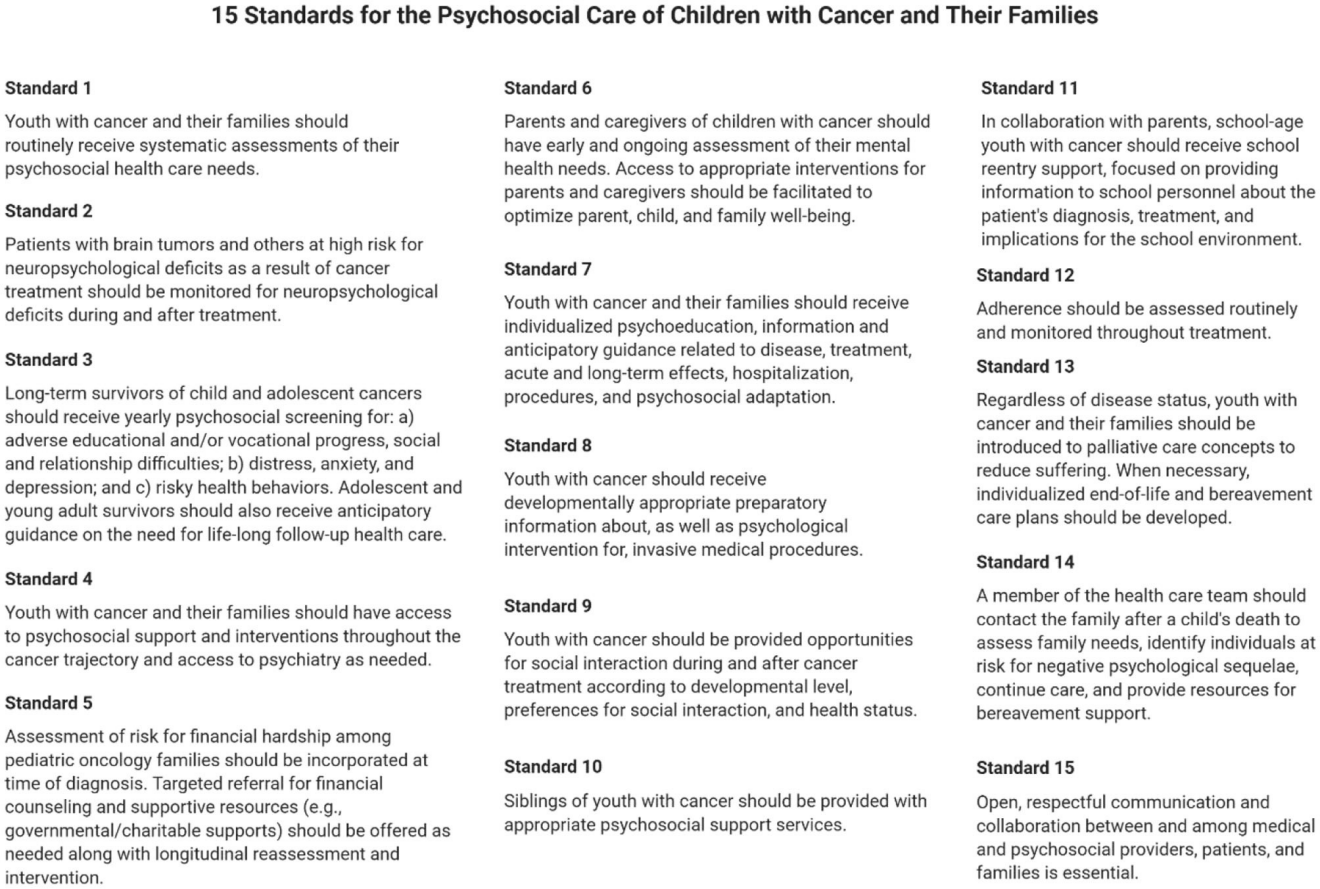
The 15 standards for the psychosocial care of children with cancer and their families, originally developed and relayed by Wiener et al. (46). Adapted from Wiener et al. (46) and created using BioRender.com.

In line with these suggestions, it is also important to address guidelines for the broader implementation of EW, video therapy, and bibliotherapy interventions on pediatric oncology wards. In 2018, Scialla et al. (45) surveyed leaders across 144 pediatric oncology programs on whether their psychosocial care practices were “state of the art.” This referred to how integrated psychosocial care was with medical care and how closely it aligned with the Standards for Psychosocial Care for Children with Cancer and Their Families (Figure 3). Unfortunately, only half of all respondents agreed that their institution's psychosocial practices were “state of the art.” Strikingly, psychosocial care (usually in the form of distraction, relaxation, and/or cognitive behavioral therapy) was also not universally provided to patients across programs—even when a problem was identified. EW, video narratives, and bibliotherapy exercises can increase the likelihood that, and shorten the time span in which, universal psychosocial care can be received. Hospitals and clinics could maintain a library of illness-related children's books and EW prompts for patients and families to use at their discretion. They might also permanently reserve a video camera in a private room, so patients and survivors can verbalize and record their deepest thoughts and feelings on difficult days. If the individual chooses, this might be done without a mental health professional or family member present so that no external pressure is felt.

Our hope is that patients and survivors of pediatric cancer may be allowed time and opportunity for self-expression and self-reflection at any time in their illness experience. EW, video narratives, and bibliotherapy exercises are easily implementable and highly accessible options for this therapeutic endeavor. If desired, they may also be a powerful supplement to the professional follow-up (*via* music therapy, play therapy, and/or talk therapy initiatives) already offered at many institutions. Ultimately, “good health lies not just in the creative expression of a patient, but in the health of a system that recognizes the benefits of integrating the extremes of human experience into a sensible, organized, hopeful, [and] perhaps beautiful structure” (47). We firmly believe that the verbal therapies reviewed here—by encouraging storytelling, narrative construction, and getting in touch with one's self—do just this.

## Author Contributions

JJ and JE: concept and design. JJ: acquisition of data, analysis and interpretation of data, and manuscript preparation. JJ, JE, and RB: manuscript revision. All authors contributed to the article and approved the submitted version.

## Conflict of Interest

The authors declare that the research was conducted in the absence of any commercial or financial relationships that could be construed as a potential conflict of interest.
